# Control of cortex development by ULK4, a rare risk gene for mental disorders including schizophrenia

**DOI:** 10.1038/srep31126

**Published:** 2016-09-27

**Authors:** Bing Lang, Lei Zhang, Guanyu Jiang, Ling Hu, Wei Lan, Lei Zhao, Irene Hunter, Michal Pruski, Ning-Ning Song, Ying Huang, Ling Zhang, David St Clair, Colin D. McCaig, Yu-Qiang Ding

**Affiliations:** 1 Key Laboratory of Arrhythmias, Ministry of Education of China, East Hospital, and Department of Anatomy and Neurobiology, Collaborative Innovation Centre for Brain Science, Tongji University School of Medicine, Shanghai 200092, China; 2 School of Medicine, Medical Sciences and Nutrition, University of Aberdeen, Foresterhill, AB25 2ZD, Aberdeen, Scotland, United Kingdom

## Abstract

Schizophrenia is a debilitating familial neuropsychiatric disorder which affects 1% of people worldwide. Although the heritability for schizophrenia approaches 80% only a small proportion of the overall genetic risk has been accounted for, and to date only a limited number of genetic loci have been definitively implicated. We have identified recently through genetic and *in vitro* functional studies, a novel serine/threonine kinase gene, *unc-51-like kinase 4* (*ULK4)*, as a rare risk factor for major mental disorders including schizophrenia. Now using the approach of *in utero* gene transfer we have discovered that Ulk4 plays a key modulatory role in corticogenesis. Knockdown of Ulk4 leads to significantly decreased cell proliferation in germinal zones and profound deficits in radial migration and neurite ramification. These abnormalities can be reversed successfully by Ulk4 gene supplementation. Ulk4 also regulated acetylation of α-tubulin, an important post-translational modification of microtubules. We conclude that Ulk4 plays an essential role in normal brain development and when defective, the risk of neurodevelopmental disorders such as schizophrenia is increased.

Schizophrenia is a chronic and disabling brain disorder with a life time risk around 1%. It is among the top 10 causes of human disability worldwide. The causes are highly variable with both genetic and environmental factors predisposing to overall risk. Although heritability is estimated at between 60–80%, the genetic architecture and the molecular mechanisms remain controversial. Current treatments are palliative and do not alter overall prognosis.

Although schizophrenia normally presents in early adult life, overwhelming evidence indicates that it has a strong neurodevelopmental component^1^,^2^. An increased predisposition has been shown to stem from various developmental insults that occur at first, or early second trimester of pregnancy. These insults often cause molecular changes which disturb the cellular architecture and neuronal connectivity and therefore interfere with brain maturation and integrity^3^. The neurodevelopmental model is based on rapidly growing knowledge from animal models^4^, environmental interactions^5^ and clinical findings including pre-existing morphological abnormalities in the brains of schizophrenia cases^6^ and behavioural changes in childhood before the onset of symptoms^7^,^8^.

It is essential to dissect out how genetic alterations predisposing to schizophrenia alter brain development and synaptic connectivity in order to understand the underlying pathogenesis. Unfortunately, despite large numbers of susceptibility genes having been proposed, only a small proportion of the overall genetic risk has been implicated robustly in human patients either by enrichment of rare gene copy number variations, or through genome-wide significant associations of common genetic variants^9^,^10^,^11^. Given the polygenic nature of schizophrenia, it is possible that manifestation of this condition represents the end point of many parallel and/or convergent pathogenic processes. Many more causative genes will need to be found and their underlying mechanisms understood before it will be possible to use genetics clinically to aid diagnosis and to partition people at risk for early prevention and for stratified therapy.

Through a meta-analysis of several large cohorts worldwide, we reported recently a serine/threonine kinase gene, *unc-51-like kinase 4* (*ULK4*), as a novel, rare risk factor for human schizophrenia, autism and major depression^12^. ULK4 belongs to the family of *unc-51*-like serine/threonine kinases which participates in a conserved pathway involving both endocytosis and axon growth^13^,^14^,^15^. Mutation of the *unc-51* gene in *C. elegans* results in stalled axon outgrowth, aberrant axonal fasciculation and abnormal accumulation of intracellular membranous structures^13^. In *Drosophila*, *Unc-51*-mediated membrane vesicle transport is pivotal in axonal and dendritic development. It aids targeted localization of guidance molecules and organelles which regulate the elongation and the compartmentalization of developing neurons and motor-cargo assembly^16^. Interestingly, although the biology of other members of *unc 51-*like kinases (ULK1-3) have been well documented^17^,^18^, the functions of ULK4 have been neglected and remain poorly understood.

Using a lentivirus-mediated gene knockdown approach, we have demonstrated that depletion of ULK4 in human neuroblastoma cells (SH5Y-SY) alters multiple signalling pathways (MAPK, p38, PKC, and JNK) associated closely with stress and schizophrenia. Consequently, the cells present abnormal cytoskeleton remodelling, reduced cell motility and reduced neuritogenesis^12^. These findings provide the first body of mechanistic evidence which potentially associates ULK4 with mental disorders.

The *in vivo* function of ULK4 is largely unknown especially in the context of neurodevelopment. Although Ulk4^−/−^ mutant mice are available, the majority of them presents with severe congenital hydrocephalus due to disrupted motile cilia protruding from ependymal cells, which frequently associates with brain hemorrhage accompanied by fibrosis, neuroinflammation and neovascularization^19^. Importantly, hydrocephalus itself profoundly impairs neurogenesis and disturbs brain development^20^,^21^,^22^,^23^,^24^. Therefore, caution is needed to interpret the phenotypes emanating from Ulk4^−/−^ mice because they may result from the hydrocephalus and not from the direct consequences of Ulk4 deletion.

In this study, we have used the technique of *in utero* gene transfer and silenced Ulk4 successfully in subsets of neural progenitors in the developing mouse cortex. We have observed a spectrum of abnormal cell behaviours including deficits in cell proliferation, neurite ramification and in the temporal sequence of neural migration. Knockdown of Ulk4 leads to decreased acetylation of α-tubulin both *in vitro* and *in vivo*. In addition, supplement of a shRNA-resistant Ulk4 cDNA reversed the observed migration and neurite arborisation abnormalities. To our knowledge, our data indicate for the first time that Ulk4 regulates key cellular and molecular events essential for normal brain development and if perturbed, may contribute to the onset of major mental disorders including schizophrenia.

## Results

### Expression profile of Ulk4 transcripts in cerebral cortex

**Figure 1:**
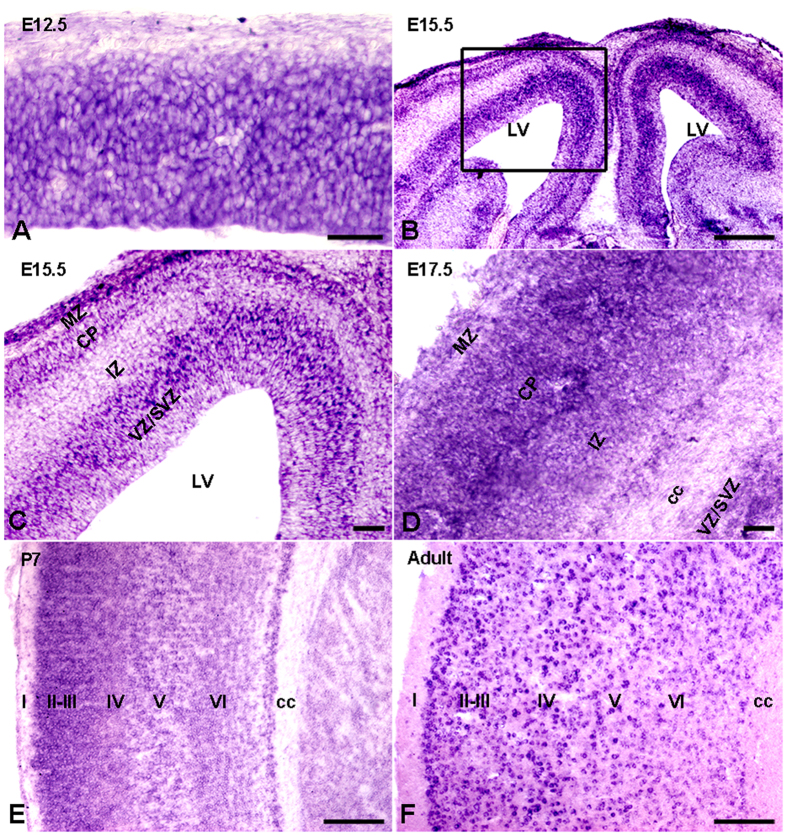
Expression profile of Ulk4 mRNA in mouse cortex. (**A**) Strong *in situ* hybridization signals were detected broadly in the whole E12.5 cortex. (**B**) In E15.5 cortex, Ulk4 mRNA was expressed highly in ventricular zone, subventricular zone and cortical plate. (**C**) Enlarged view of the boxed region in (**B**). (**D**) Ulk4 mRNA was expressed widely in E17.5 cortex, with a preferable location in upper layers. (**E,F**) Similar to the expression profile of E17.5 cortex, Ulk4 mRNA was expressed extensively in the cortex at postnatal day 7 and 4 month with a predominant location in layers II–IV. cc, corpus callosum; VZ, ventricular zone; SVZ, subventricular zone; IZ; intermediate zone; CP, cortical plate; MZ, marginal zone. I–VI, sublayers of cortex. Bars = 100 μm in (**B,E,F**) and 20 μm in (**A,C,D**).

Previously, we reported abundant neuronal expression of ULK4 protein in adult human and mouse brains by immunohistochemistry^12^. To understand further the precise function of Ulk4 in cortical development, we synthesized specific riboprobes and performed *in situ* hybridization in both embryonic and postnatal mouse brains. Ulk4 transcripts were detected widely in the E12.5 cortex, when active cortical neurogenesis takes place and brain lamination arises (Fig. 1A). In E15.5 cortex, abundant hybridization signals were observed in the ventricular zone (VZ), subventricular zone (SVZ) and cortical plate, but only a weak signal was evident in the intermediate zone (Fig. 1B,C). This distribution pattern highlights the potential regulatory roles of Ulk4 in both neural progenitors and post-mitotic neurons which have exited the cell cycle and initiate migration and differentiation. In E17.5 cortex, dense hybridization signals were located in cortical plate (CP) and were more prominent than in the intermediate zone and SVZ/VZ regions (Fig. 1D). At postnatal day 7, Ulk4 transcripts were distributed extensively in all cortical layers (Fig. 1E) and in adulthood (Fig. 1F), which mirrors our previous immunohistochemical findings^12^. Hybridization with sense probes also was carried out and no specific signals were detected (Supplementary Fig. S1). These results show wide and dynamic Ulk4 expression in both developing and postnatal cortex which strongly supports a modulatory role of Ulk4 in corticogenesis and functional maturation.

### Knockdown of Ulk4 at E15.5 significantly affected corticogenesis

In view of the intense expression of Ulk4 mRNA in germinal zones of developing cortex, especially at E12.5 and E15.5 (Fig. 1A–C), we speculate that Ulk4 may regulate the proliferation of neural progenitors and thus control the expansion of stem cell pools. To obtain direct evidence for this hypothesis, we analysed the influence of Ulk4 knockdown through an *in utero* gene-transfer technique via electroporation.

**Figure 2:**
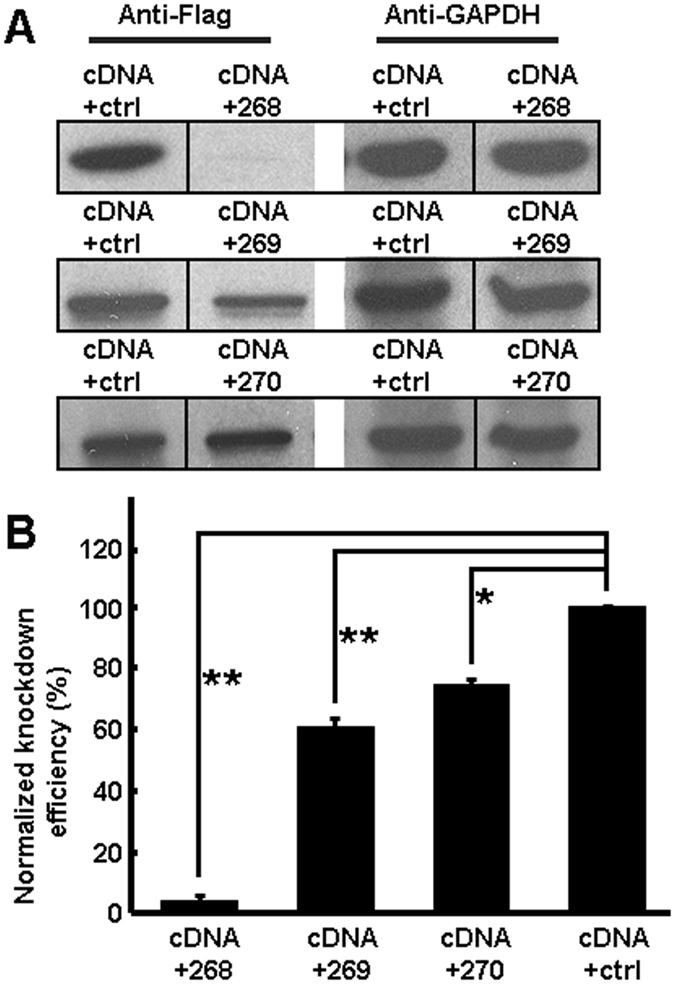
Validation of Ulk4 shRNA268, 269 and 270. (**A**) Western blotting with anti-Flag antibody shows that shRNA268 mediates the strongest knockdown of Ulk4 whilst shRNA270 displays a mild effect. (**B**) Normalized knockdown efficiency of the three shRNAs (n = 3 for each). *p < 0.05, **p < 0.01, two-tailed student’s *t* tests. Error bars indicate mean ± S.E.M.

We first tested the knockdown efficiency of Ulk4 shRNA268, 269 and 270 by co-transfection with Ulk4 full-length cDNA (tagged with c-Myc and DDK) in HEK293 cells. Two days after transfection, cells were lysed and blotted with anti-Flag. The results showed that among the three Ulk4 shRNAs, 268 presented the strongest knockdown capacity whilst 270 displayed the weakest effect (Fig. 2A,B). Blotting with anti-c-Myc also demonstrated similar knockdown capacity of the three shRNAs (Supplementary Fig. S2). Therefore, shRNA268 and 269 were chosen for functional analysis.

**Figure 3:**
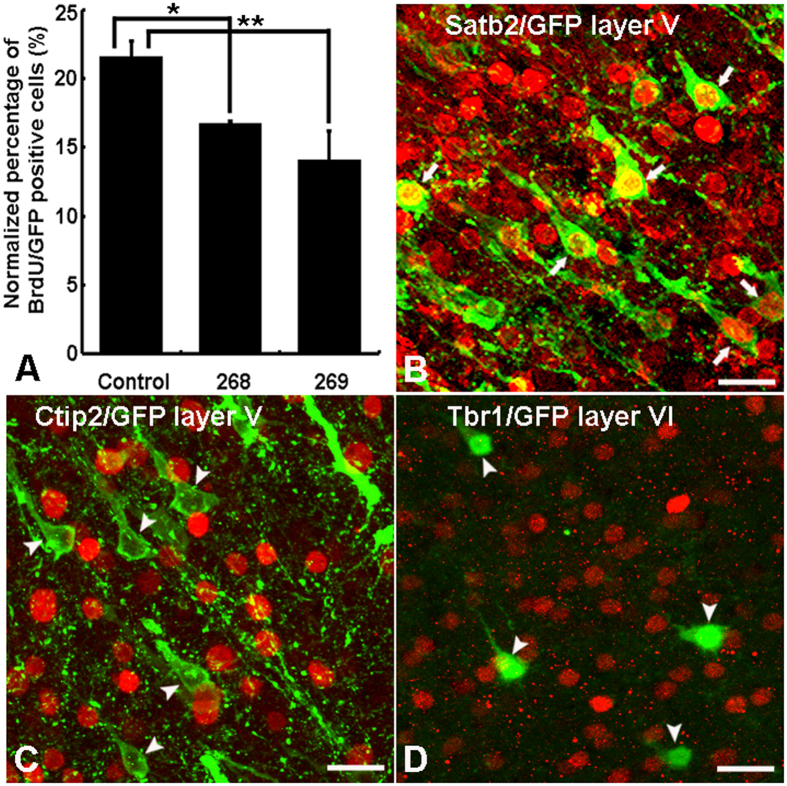
Ulk4 knockdown inhibits proliferation of cortical neural stem cells but does not change cell fate determination. (**A**) Both shRNA268 and 269 inhibit the proliferation of neural precursors. *p < 0.05, **p < 0.01. Error bars indicate mean ± SD. (**B**–**D**) The majority of the “trapped” GFP neurons, if not all, in the deeper layers of knockdown brains still express Satb2 (**B**, white arrows), instead of Ctip2 (**C**, white arrowheads) and Tbr1(**D**, white arrowheads). Bars = 20 μm.

Panels of plasmids containing shRNA268 or 269 together with GFP were injected into lateral ventricles of E15.5 mouse brains prior to *in utero* electroporation. The embryonic brains were collected on the next day (E16.5) one hour after BrdU pulse injection. Immunostaining with anti-Ulk4 antibody demonstrated a successful Ulk4 targeting (Supplementary Fig. S3). To estimate the proportion of transfected cells in S phase, immunostaining with anti-BrdU (Supplementary Fig. S4) was performed and the number of GFP/BrdU double-labelled cells was counted. The expression ratio was calculated by normalization against the total number of GFP-containing cells. Our results suggest that both Ulk4 shRNA268 group (n = 3, *p* < 0.02) and 269 group (n = 3, *p* < 0.001) have a significantly lower percentage of GFP/BrdU double-labelled cells compared with control group (n = 4) (Fig. 3A). These results strongly indicate that silencing Ulk4 in the developing brain significantly inhibits neural progenitor proliferation.

### Knockdown of Ulk4 at E15.5 disturbed neuronal migration and neurite arborisation substantially

**Figure 4:**
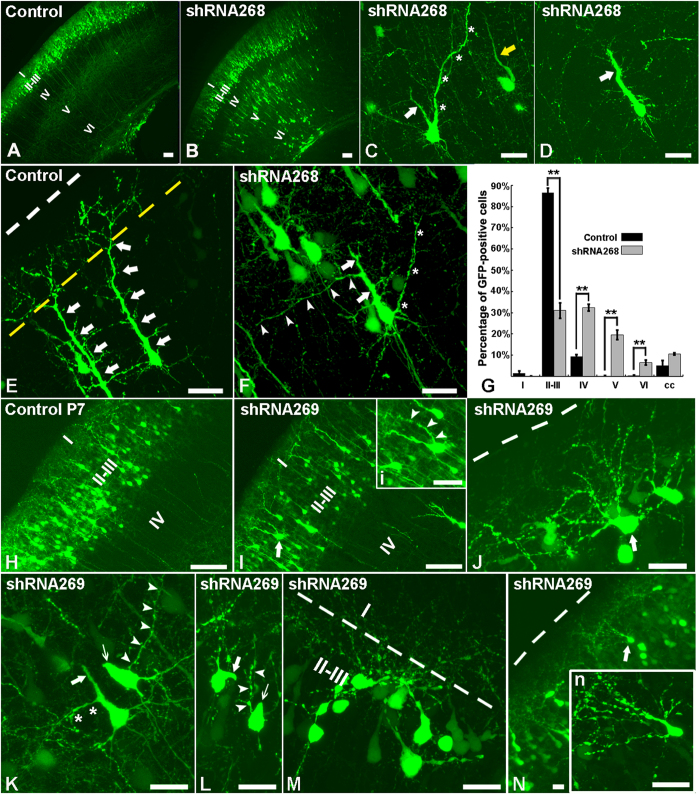
Silencing Ulk4 massively perturbs radial migration and neurite arborization. (**A**) In the control cortex, almost all the neurons traced with GFP at E15.5 have migrated successfully into layers II–III at P7; (**B**) Instead of being present in superficial layers, large amounts of GFP-traced cells are “trapped” in cortical layers IV–VI in shRNA268 knockdown groups. (**C,D,F**) Ulk4 knockdown with shRNA268 dramatically reshapes neurite branching. Most of the mislocated GFP neurons bear a stalled apical dendrite (white arrows) with some having an ectopic apical dendrite (**C**, asterisks and yellow arrow; **F**, asterisks) or long secondary dendritic branching (**F**, white arrowheads). (**E**) In the control brains, GFP-positive pyramidal neurons develop a predominant apical dendrite (white arrows) which ends and elaborates in layer I. White broken line shows the pial surface and the yellow broken line delineates the boundary between lamina I and II. (**G**) Compared with control brains (black columns), there are significantly more cells in deeper cortical lamina and subtantially less cells in layers II–III in the shRNA268 knockdown cortex (gray columns). *p < 0.05. **p < 0.01. (**H,I**) Compared with control brains (**H**), shRNA269 leads to dispersed layers II–III which contain loosely-packed GFP cells (**I**). White arrow in I shows a neuron with two apical dendrites which is also enlarged in inset i. (**J**–**N**) Confocal images of abnormal cell morphology in shRNA269 knockdown brains. Some cells develop multiple dendrites directly from the soma with (**N**, white arrow, and inset n) or without the predominent apical dendrite (**J,** white arrow). Inset n is the enlarged view of the white arrowed neuron in **N**. In addition, many GFP-positive cells have stalled (**K,L**, white arrows) or aborted (**K,L**, slim white arrows) apical dendrites which are frequently replaced by ectopic apical dendrites (**K,L**, white arrowheads) or long secondary dendritic branching (K, asterisks). (**M**) Clusters of GFP-postive cells are disoriented and do not present typical polarity of pyramidal neurons (aligned perpendicularly to brain surface). Broken white lines in (**J,N**) indicate the brain surface and the boundary between layers I and II in (**M**). Bars = 100 μm in (**A,B**), 20 μm in (**C**–**N**) and associated insets.

The strong expression of Ulk4 transcripts in the post-mitotic neurons prompted us to investigate the influence of Ulk4 on radial migration, a precisely-controlled process by which neurons move from their site of origin to the final laminar position. To address this question, we examined the distribution of GFP-traced cells in the P7 brains which underwent electroporation at E15.5. In the control group, the majority of neurons (born on E15.5) have completed migration and settled compactly in layers II–III (Fig. 4A), a highly vulnerable target region in schizophrenia. Strikingly, in the shRNA268 knockdown brains, GFP-positive neurons were located in loose arrays within layers II–III. In addition, many neurons were scattered along the migration route, and many others appeared trapped in the deeper cortical lamina close to the corpus callosum (Fig. 4B). We counted the number of GFP-positive cells in each individual layer and performed statistical comparison. The results show that compared with control brains (n = 3), knockdown brains contained significantly less GFP-positive cells in layers II–III (n = 3, *p* < 0.001), but high proportions of these cells in deeper layers (IV–VI) (*p* < 0.001) (Fig. 4G), indicating a dramatically delayed neuronal migration.

To further validate the identities of neurons with Ulk4 knockdown, we carried out immunostaining by using antibodies against specific transcription factors including Satb2 (strongly expressed in cortical neurons, especially layers II–III), Ctip2 (commonly used for layer V/VI) and Tbr1 (a marker for layer VI). Most of these GFP-traced cells in layers V–VI also expressed Satb2 (Fig. 3B, white arrows), however, almost no GFP-positive cells in deeper cortical layers displayed positive staining for either anti-Ctip2 (Fig. 3C, white arrowheads) or anti-Tbr1 (Fig. 3D, white arrowheads). Similarly, the GFP-positive cells in layer II–III also expressed Satb2, but did not display positive staining for both anti-Ctip2 and anti-Tbr1 (Supplementary Fig. S5). This result suggests that Ulk4 knockdown does not change the fate specification of these “trapped” GFP cells which are geographically “ectopic” layer II/III neurons.

**Figure 5:**
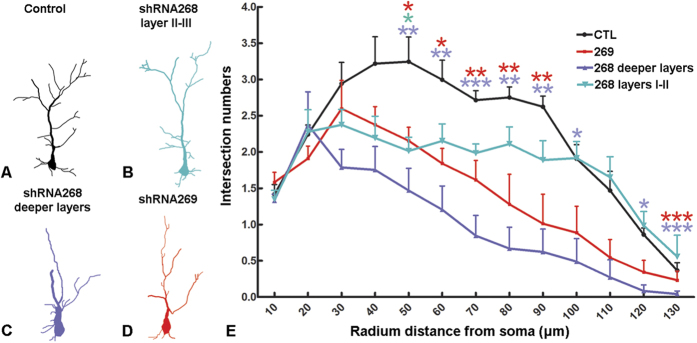
Ulk4 Knockdown significantly reduces neurite ramification. (**A–D**) 2-D reconstruction of GFP-labelled neurons in control (**A**), layers II–III of shRNA268 knockdown cortex (**B**), deeper layers of shRNA268 knockdown cortex (**C**) and shRNA269 knockdown cortex (**D**). (**E**) *Sholl* analysis shows that neurons in Ulk4 knockdown cortex present poorly-developed neurite arborization. Forty-one cells were reconstructed from 6 control brains, 30 cells were from layers II–III of 3 shRNA268 brains, 33 cells from deeper layers of 4 shRNA268 brains and 23 cells from 3 shRNA269 brains; Two-tailed student’s *t* tests. **p < 0.01, *p < 0.05.

In separate experiments, electroporation at E15.5 with low concentration of GFP (0.1μg/μl), which highlights fewer GFP-expressing neurons, was used to facilitate the morphological reconstruction of the GFP-expressing neurons by z-stack confocal scanning. In control cortex, the majority of cells in layers II–III presented the typical shape of pyramidal neurons, with a long apical dendrite branching out in an apical tuft which terminates in layer I (Fig. 4E). In the shRNA268 knockdown brains, most of the GFP-positive cells in the deeper layers developed stalled apical dendrites and had a poorly-formed apical tuft (Fig. 4C,D,F, white arrows). Intriguingly, some have an “ectopic” apical dendrite (Fig. 4C,F, white asterisks) and long secondary dendritic branching (Fig. 4F, white arrowheads) or an apical dendrite which did not stick out from the apex of the cell body (Fig. 4C, yellow arrow). We reconstructed and compared the dendritic trees between control and knockdown neurons using confocal microscopy. As expected, the GFP-labelled neurons in knockdown brains, regardless of position, presented significantly reduced dendrite network complexity (Fig. 5A–C,E, 41 cells from 6 control brains, 33 cells in layers II–III from 4 shRNA268 brains, 30 cells in deeper layers from 3 shRNA268 brains; **p < 0.01, *p < 0.05). This result is also in agreement with our previous observation that ULK4 deletion leads to a remarkable impairment of neuritogenesis in SHSY-5Y cells^12^.

We carried out the same sets of studies in shRNA269 knockdown brains in order to rule out any “off-target” effects. Although there was no significant migration delay in the deeper layers of the P7 knockdown brains (Fig. 4I), layers II–III however were slightly dispersed and full of loosely packed and aberrantly aligned GFP-positive neurons (Fig. 4H,I). Similarly, many of these cells had ectopic (Fig. 4I, inset *i*; Fig. 4K,L, white arrowheads), stalled (Fig. 4K, white arrows) or aborted apical dendrites (Fig. 4K,L, slim white arrows) and long secondary branching (Fig. 4K, asterisks), as was observed in the shRNA268 knockdown brains. Some cells had multiple apical dendrites with (Fig. 4N, white arrow, and inset *n*) or without a clearly predominant one (Fig. 4J, white arrow). In addition, areas containing dis-oriented cell populations which have lost the polarity of pyramidal neurons were apparent (Fig. 4M). *Sholl* analysis again showed that the knockdown cells had poorly-developed dendritic arborization (Fig. 5A,D,E, 37 cells from 6 shRNA269 brains).

### Knockdown of Ulk4 leads to decreased acetylation of α-tubulin both *in vitro* and *in vivo*

**Figure 6:**
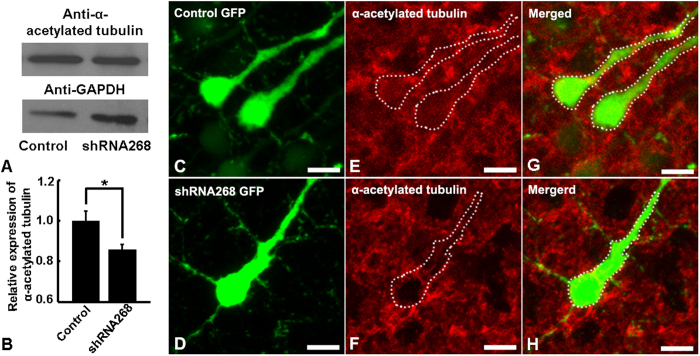
Ulk4 knockdown leads to decreased acetylation of α-tubulin both *in vitro* and *in vivo*. (**A,B**) Primary cultured neurons transfected with shRNA268 display less α-acetylated tubulin (n = 3). Two-tailed student’s *t* tests. *p < 0.05. (**C–H**) Compared with neurons transfected with control shRNA (**C,E,G**), cortical neurons incorporated with shRNA268 (**D,F,H**) displayed reduced expression of α-acetylated tubulin (in red) *in vivo*. Bars = 25 μm in (**C–H**).

How Ulk4 silencing leads to abnormal corticogenesis remains unclear. In our previous study, depletion of ULK4 in SHSY-5Y cells remarkably decreased acetylation of α-tubulin^12^. Intriguingly, reduced tubulin acetylation could result in profound deficits in radial migration and neurite ramification during brain development^25^,^26^ and disrupt hippocampal formation^27^. We therefore postulate that acetylated α-tubulin could be a component molecule of Ulk4 signalling which modulates corticogenesis. To test this hypothesis, we transfected primary cultured mouse cortical neurons with control and shRNA268 plasmids. The transfection efficiency was 76% and 74% respectively with no statistically significant difference (*p* > 0.05). We then determined the expression of acetylated α-tubulin by western blotting. Our results indeed showed that shRNA268 significantly reduced the expression of acetylated α-tubulin in the cultured neurons (n = 3, *p* < 0.05, Fig. 6A,B). In P7 brains, Ulk4 knockdown resulted in consistently diminished expression of acetylated α-tubulin (Fig. 6C–H). The reduction of acetylated α-tubulin also was confirmed by imaging analysis in both primary cultured neurons and brain sections (Supplementary Fig. S6). Similarly, shRNA269 also led to reduced expression of acetylated α-tubulin in P7 mouse brains (Supplementary Fig. S7). These results suggest that acetylated α-tubulin may contribute at least partially, to the altered corticogenesis caused by Ulk4 knockdown.

### Supplementation of Ulk4 successfully reversed the effects of Ulk4 silencing *in vivo*

**Figure 7:**
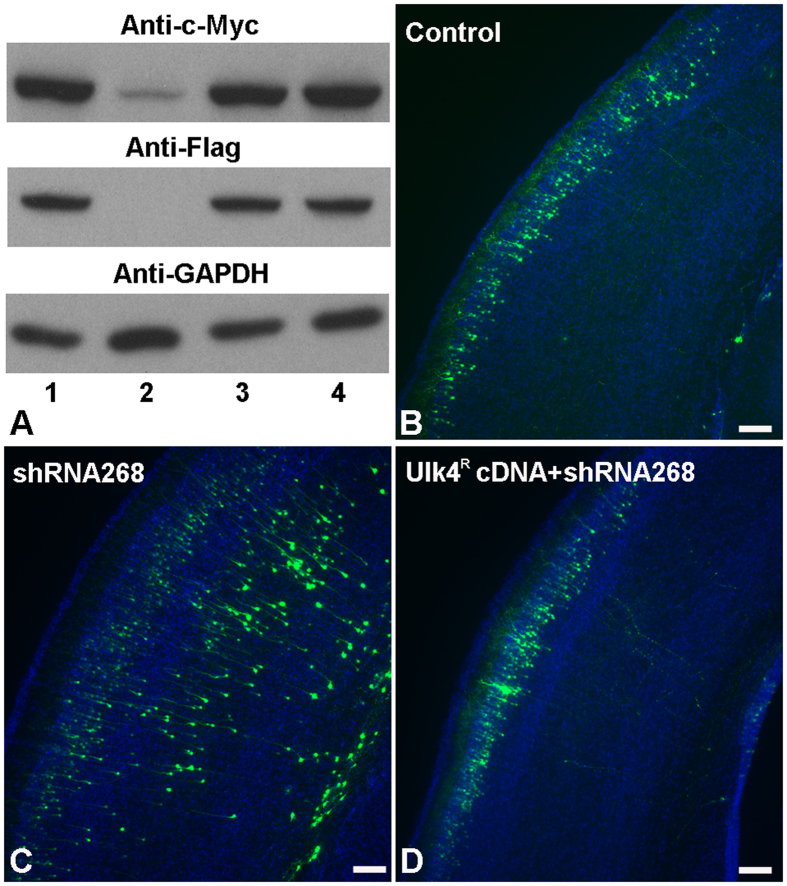
Ulk4^R^ cDNA successfully rescues the defective radial migration mediated by shRNA268. (**A**) Validation of Ulk4^R^ cDNA. Panels of plasmids containing different DNA constructs were co-transfected into HEK293 cells. Western blotting with anti-c-Myc (top row) and anti-flag (mid-row) indicated that Ulk4^R^ cDNA successfully restores the expression of Ulk4. Lane 1: co-transfection with plasmids containing control shRNA and Ulk4 cDNA; Lane 2: co-transfection with Ulk4 cDNA and shRNA268; Lane 3: co-transfection with control shRNA and Ulk4^R^ cDNA; Lane 4: co-transfection with plasmids containing shRNA268 and Ulk4^R^ cDNA. (**B–D**) Compared with the control group (**B**), the defective radial migration caused by shRNA268 (**C**) can be successfully rescued by Ulk4^R^ cDNA (**D**). Bars = 100 μm in (**B**–**D**).

To further validate that the deficits described above were directly caused by Ulk4 knockdown, we designed and cloned shRNA268-resistant Ulk4 cDNA (Ulk4^R^ cDNA) by introducing 7 point mutations into Ulk4 full length cDNA within the targeting locus without alteration of the amino acid sequence. We chose shRNA268 because of its potent knockdown ability which leads to the overt phenotypes *in vivo* (Fig. 4B–F). To determine the resistant capacity of Ulk4^R^ cDNA against shRNA268, we transfected both of these two constructs into HEK293 cells and then determined the Ulk4 expression by western blotting. Our results showed that the reduced expression of Ulk4 caused by shRNA268 was recovered successfully by Ulk4^R^ cDNA (Fig. 7A).

**Figure 8:**
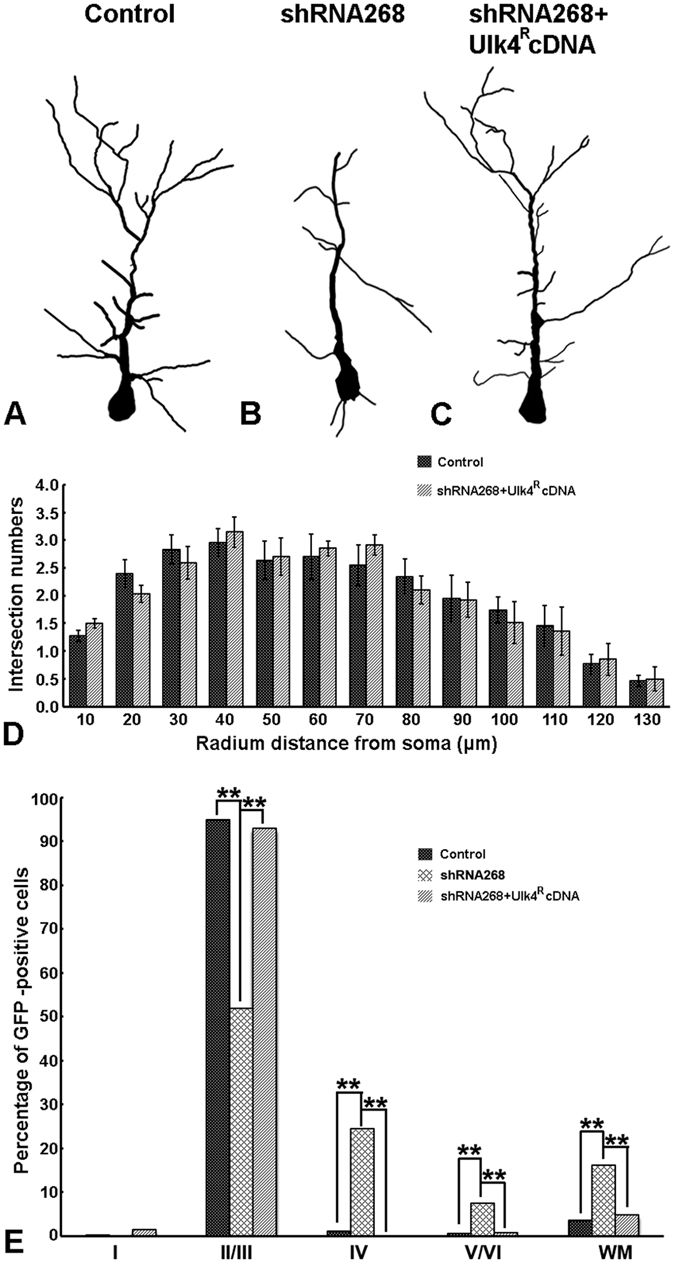
Ulk4^R^ cDNA successfully restores the normal neuronal ramification and migration. (**A–C**) Schematic drawing of neurite ramification of neurons electroporated with control (**A**), shRNA268 (**B**) and shRNA268+Ulk4^R^ cDNA (**C**). (**D**) Comparable dendritic pattern for neurons electroprated with control shRNA or shRNA268+Ulk4^R^ cDNA by *Sholl* analysis. Forty-one cells were included from 6 control brains and forty-three cells were analysed from 4 Ulk4^R^ cDNA brains. Two-tailed student’s *t* tests were performed and no significant difference was found at any examined radial distance. (**E**) Control shRNA and shRNA268+Ulk4^R^ cDNA groups show comparable percentage of GFP-tracked cells in each individual cortical sublayers.

To determine the effects of Ulk4^R^ cDNA *in vivo*, panels of expression plasmids including control, shRNA268 or shRNA268+Ulk4^R^ cDNA were delivered into lateral ventricles of E15.5 mouse brains and *in vivo* gene transfer performed and sections of P7 brains were examined to trace the positioning of these transfected cells. Compared with the control group (Fig. 7B), almost all GFP-tracked cells in the Ulk4^R^ cDNA group were located in layers II–III (Fig. 7D). This observation was confirmed further by statistical analysis of the percentage of cell numbers in individual sublayers (Fig. 8E; n = 3 for each group, **p < 0.01). These results suggest that this construct successfully resumed normal migration and settlement of layers II–III neurons which had been perturbed by shRNA268 (Fig. 7C). We further reconstructed and compared the arborisation pattern of apical dendrites between the two groups in subsequent electroporation using lower concentration of GFP. *Sholl* analysis showed that dendrite branching deficits mediated by shRNA268 have been rescued effectively by Ulk4^R^ cDNA (Fig. 8A–D, forty-one cells were included from 6 control brains and forty-three cells were from 4 Ulk4^R^ cDNA brains). Our results strongly indicate that the phenotypes caused by Ulk4 knockdown are specific and can be restored successfully upon Ulk4 overexpression.

## Discussion

Schizophrenia is a severe neuropsychiatric disorder with significant genetic signatures. Although the precise aetiology is debatable, the genetic alterations are believed, alone or in combination, to perturb the process of normal brain development and maturation, which in part causes this condition. Considering its polygenic nature, identification and validation of novel risk factors will provide new insights into this disease. We were the first to identify ULK4 as a novel rare risk factor of schizophrenia^12^. Here, we provide the first body of evidence that Ulk4 is involved closely in the regulation of corticogenesis and that disrupted Ulk4 expression leads to neurodevelopmental abnormalities which could perturb brain development and function and contribute to mental disorders with a significant neurodevelopmental component.

Corticogenesis is a precisely controlled process which is vulnerable to a variety of genetic and environmental insults. In our study, the strong expression of Ulk4 transcripts in neurogenic regions of embryonic mouse brains suggests a functional involvement in corticogenesis. Coincidentally, a recent *in situ* hybridization study has shown abundant Ulk4 transcripts in ventricular and subventricular zones in *Xenopus laevis*. Further immunohistochemical staining also revealed localization of Ulk4 in neural stem cells, radial glia and cells with neural commitment^28^. This morphological evidence highlights important regulatory roles for Ulk4 during neurogenesis. Indeed, in our study, knockdown of Ulk4 in embryonic brains caused reduced proliferation of neural stem cells as well as aberrant radial migration and positioning of projecting neurons. These alterations could be restored however upon replenishment of the Ulk4 protein. Interestingly, de-regulated neurogenesis is shared by many other generalized candidates for schizophrenia such as *Neuregulin* (NRG1) and *Disrupted-in-schizophrenia 1* (DISC1). Aberrant NRG1-ErbB4 signalling not only alters the proliferation of neuronal progenitors *in vitro*^29^ and *in vivo*^30^, but also interferes with fundamental processes of neuronal development^31^, including the radial migration of glutamatergic neurons^32^ and the tangential migration of GABAergic neurons^33^. *Disc1* not only modulates embryonic^34^ and adult neurogenesis^35^, but also governs the production and settlement of glutamatergic and GABAergic neurons through radial^36^,^37^,^38^ and tangential migration^39^.

Dysfunction of proper GABAergic inhibition in the cerebral cortex underlies at least part of the pathophysiological process of schizophrenia both in mice and humans^6^,^40^. Although we have no direct evidence to support the modulatory roles of Ulk4 in the origin and migration of GABAergic interneurons, we have observed strong *in situ* signals of Ulk4 transcripts in medial and lateral ganglionic eminences of the telencephalon, which accommodates GABAergic interneuron precursors and facilitates tangential migration. Moreover, we also observed wide Ulk4 expression in GAD67-postive neurons in both human and mouse brains^12^. Concordantly, Ulk4 heterozygous mice present reduced expression of GAD67 and components of GABA_A_ receptor subunits (β1, ɛ, δ, ρ2) in amygdala and display anxiety-like behaviour^41^. Interestingly, *CTNNB1*, an essential component in the WNT signalling pathway and a 3′-flanking gene of *ULK4*, participates in hippocampal network regulation and contributes to the decreased GAD67 expression in both schizophrenia and bipolar patients^42^. Therefore, it is likely that Ulk4 also provides instructive cues to coordinate the migration and functional integrity of interneurons though the precise process needs further investigation.

Proper neurite arborisation is important not only in establishing appropriate synaptic contacts but also in mediating neuronal plasticity which underlies important aspects of behaviour. Impaired neuronal connectivity and synaptic plasticity have been considered as an endo-pathogenesis of schizophrenia. In the present study, we used two shRNAs (268 and 269) which recognize distinct DNA coding sequence and display different knockdown capacity (Fig. 2) and phenotypes. While shRNA268 significantly inhibits radial migration but moderately affects cell proliferation, shRNA269 strongly impedes cell division and only slightly affects radial migration (Figs 3 and 4). However, both of them robustly interfere with neurite growth and arborisation (Fig. 5). In both groups, the pyramidal neurons in layers II–III consistently displayed abnormal neurite branching including stalled apical dendrites and poorly developed dendritic tree. Similarly, defective neuritogenesis also was observed in SHSY-5Y cells with Ulk4 depletion^12^. Due to technical limitation, we could not perform the same analysis in adult mice as GFP may be degraded over a long period of time and a conditional knockout paradigm could be a better option. Nevertheless, imaging studies have shown a reduced brain volume and progressive grey matter loss in schizophrenia patients even before the onset of the disease, implicating a contribution of aberrant neurodevelopment^43^. This reduction is largely due to diminished neuropil and smaller neuronal sizes rather than decreased cell numbers^6^,^44^. Our results support the involvement of Ulk4 in neurodevelopment and synaptic connectivity which is compatible with current neuro-imaging and post-mortem studies^3^,^6^,^45^,^46^,^47^,^48^. On the other hand, we have observed ULK4 deletion in Caucasian autism populations^12^. Ou *et al*. reported that ULK4 genetic variants strongly associate with Han Chinese autism and affect ULK4 expression in the prefrontal cortex of post-mortem human brains^49^. In the BBGRE cohort, 7/5891 patients exhibit ULK4 deletion and 6 deletions are unique, intragenic and non-related. These patients manifest a range of developmental disorders including developmental delay, behavioural problems, severe learning difficulties and severe language delay^50^. We assume that ULK4 may be involved in these conditions through common pathological alteration such as deficits in dendrite formation and neural network described by us.

ULK4 belongs to the *unc-51*-like serine/threonine kinase family which participates in a conserved pathway involving both endocytosis and axon growth^13^,^14^,^15^. Due to insufficient studies, the precise function and relevant pathways of Ulk4 remain unclear though the other family members are all well-studied. Recent GWAS studies suggest that Ulk4 is a risk locus for multiple myeloma^51^ and inter individual diastolic blood pressure variation^52^. In mice, targeted deletion of Ulk4 leads to significantly shorter motile cilia on respiratory and ependymal epithelia. These mice display a series of ciliopathy-related phenotypes including respiratory inflammation, serious congenital hydrocephalus and even otitis media^19^. During the revision of this manuscript, Liu *et al*. reported that Ulk4^−/−^ mice present a thinner cortical layers II–III owing to a decreased proliferation of NSCs at E15.5^50^. However, it is not ideal to investigate the proliferation of NSCs utilizing Ulk4^−/−^ mice because the accumulated cerebrospinal fluid (CSF) during hydrocephalus robustly disrupts the cell cycle^23^ and inhibits proliferation of NSCs^21^. CSF also contains many neurotrophic or growth factors such as bone morphogenetic protein, fibroblast growth factor (FGF), insulin-like growth factor, sonic hedgehog, retinoic acid, brain derived neurotropic factor (BDNF) and WNT^24^. These circulating factors regulate the mitosis of NSCs closely and their levels are altered in both animal models and human hydrocephalus patients^20^,^22^,^24^. For example, bFGF is expressed in the ventricular epithelium and choroid plexus^53^,^54^. Immuno-depletion of CSF bFGF in embryonic brains reduces the proliferation of neural progenitors^55^,^56^. Ventricle administration of bFGF induces hydrocephalic brain morphology and aberrant differentiation of neurons in the cerebral cortex^54^. BDNF promotes NSCs to exit the cell cycle, down-regulates the expression of PAX6, an essential transcription factor for generation of layer II–III neurons, and determines neuronal laminar fate in the developing mouse cerebral cortex^57^. Interestingly, in rats with congenital hydrocephalus, the expression of BDNF is increased in ventricular zones where NSCs reside^20^. Therefore, utilizing Ulk4 KO mice for neurodevelopmental studies inevitably will introduce many new variances and ignoring the influence of these variances may lead to biased judgement and conclusions.

One of the most striking findings in our study is the reduced acetylation of α-tubulin, which can be replicated consistently in SHSY-5Y cells^12^, primary cultured neurons and brain sections upon Ulk4 knockdown. Tubulin acetylation is an important post-translational modification which not only stabilizes microtubules but is required for polarity establishment and directional migration. In cultured neurons, tubulin acetylation is important for polarized protein trafficking and regulates cell shape and neurite outgrowth^27^,^58^. In mice, decreased acetylation of α-tubulin results in abnormal hippocampal development^27^, profound impairment of radial migration and the branching of projecting neurons in cortex^25^,^26^. Proper acetylation of α-tubulin has been reported in various neurological conditions. For example, α-tubulin acetylation is reduced in brains with Huntington’s disease, and enhancement of acetylation improves the recruitment of dynein and kinesin-1 to microtubules and increases the flux of vesicles and subsequent release of brain-derived neurotrophic factor^59^. Similarly, in Parkinson’s disease, increased tubulin acetylation alleviates α-synuclein-mediated neurotoxicity^60^. Histone deacetylase 6 (HDAC6) functions in many cellular events by deacetylating non-histone proteins including α-tubulin and HDAC6 inhibition could generate antidepressant-like properties with improved brain bioavailability^61^. In line with this observation, HDAC6^−/−^ mice exhibit less anxiety and antidepressant-like behaviour^62^. In addition, HDAC6 mutation could increase α-tubulin acetylation and rescue human tau-induced microtubule defects in *Drosophila*^63^. In our experiments, we observed reduced acetylation of α-tubulin and overt deficits in cell migration and neurite arborisation. We assume that these phenotypes would be fundamental for Ulk4-relevant neurodevelopmental diseases and further studies including behavioural tests would be important.

On the other hand, acetylated α-tubulin is an important cytoskeleton component of the axoneme, the core structure of cilium. The cilium is an antenna-like small organelle which exists in most mammalian cell types and when defective, leads to a suite of inherited diseases known as ciliopathies. It is not clear whether Ulk4 null knockout mice have reduced acetylated α-tubulin, however, they do present shorter and disorganized motile cilia on respiratory epithelium and on ependymal cells lining the ventricles indicating that they are dysfunctional^19^. Although no imaging studies are available from patients with ULK4 mutation, imaging data has suggested increased global or regional cerebrospinal fluid (CSF)^45^,^46^,^64^,^65^ and larger 3^rd^ and lateral ventricles^6^ in schizophrenia patients. Thus, it would be interesting to investigate how defective acetylated α-tubulin caused by Ulk4 disruption impairs motile cilium elongation and the wave-like beating which propels the directional CSF flow. We assume that Ulk4-relevant diseases may be linked with ciliopathies, neurodevelopmental disorders and psychosis.

In summary, we have provided the first *in vivo* evidence that ULK4 is implicated in fundamental cellular and molecular processes during corticogenesis. Ulk4 disruption leads to multiple abnormalities common to multiple neurodevelopmental disorders, including schizophrenia and autism.

## Methods

### Plasmids and DNA constructs

Three individual plasmids harbouring different mouse Ulk4 shRNAs were purchased from Sigma (TRCN0000328268: 5′-CCGGCTGCGAAGATTATCGAGAATGCTCGAGCATTCTCGATAATCTTCGCAGTTTTTG-3′; TRCN0000328269: 5′-CCGGGAAGCAGGACTGTCGTATATACTCGAGTATATACGACAGTCCTGCTTCTTTTTG-3′; TRCN0000328270: 5′-CCGGAGCTGAATGAATCCATATTTCCTCGAGGAAATATGGATTCATTCAGCTTTTTTG-3′). These plasmids contained the backbone of TRC2-pLKO-puro vector with the insertion of the corresponding shRNA sequence. They were abbreviated as shRNA268, 269 and 270 respectively in the subsequent procedures. A control plasmid was also from Sigma which contains a non-specific sequence for any mammalian gene (SHC002; 5′-CCGGCAACAAGATGAAGAGCACCAACTCGAGTTGGTGCTCTTCATCTTGTTGTTTTT-3′). The knockdown efficiency of these three shRNAs was assessed by co-transfection with full-length Ulk4 cDNA (MR217918, Myc-DDK-tagged, Origene) and determined by western blotting. The shRNA268-resistant mouse Ulk4 cDNA (Ulk4^R^ cDNA) was generated from Ulk4 cDNA by introducing seven nucleotide mutations in the shRNA268 target region without changing the coded amino acids. Its ability against shRNA268 was evaluated by co-transfection in HEK293 cells followed by western blotting.

### In Utero Electroporation

Time-mated pregnant mice (E15.5) were anaesthetized and embryos were manipulated surgically as described previously^66^. Plasmids containing Ulk4 shRNAs 268 or 269 (0.5 μg/each) were injected into the lateral ventricles of the embryonic brains through glass micropipettes. CAG-EGFP was also injected (1 μg/μL) simultaneously in order to trace the successfully transfected cells. To reverse the phenotypes caused by Ulk4 knockdown, Ulk4^R^ cDNA was co-injected together with shRNA268. To label neurons relatively isolated from other transfected cells, the concentration of CAG-EGFP was reduced to 0.1 μg/μl. Five square electric pulses (30 V) were delivered through the uterus at 1s intervals with forceps-type electrodes while the uterus was kept wet with saline. The female mice were then divided into two groups. In group one, they were culled one day after electroporation and the embryonic brains were harvested (E16.5) one hour after a single pulse of BrdU injection (100 mg/kg). In group two, the pup brains were collected on postnatal day 7 (P7) for histochemical examination.

### Cell culture, western blot and immunoprecipitation

HEK293 cells were maintained in Dulbecco’s modified Eagle’s medium (DMEM) (Gibco) containing 10% fetal bovine serum (Invitrogen). To determine the knockdown efficiency of Ulk4 shRNAs, cells at 80% confluence were transfected with plasmids containing either control (1 μg) or Ulk4 shRNAs (268 or 269, 1 μg/each) combined with full length mouse Ulk4 cDNA (1 μg) using jetPRIME transfection reagent (Source Biosciences, UK). To test the function of Ulk4^R^ cDNA, full length Ulk4 cDNA was replaced by Ulk4^R^ cDNA (1 μg) and co-transfected with shRNA268 for 2 days. For western blotting, proteins were extracted with RIPA buffer containing complete protease and phosphatase inhibitors (Roche). Equal amounts of protein were fractionated by SDS-PAGE, transferred to nitrocellulose membranes and blotted with primary antibodies. The following primary antibodies were used: rabbit anti-c-Myc (1:2000, Sigma), rabbit anti-Flag (1:2000, Sigma), mouse anti-Flag (1:2000, Sigma), mouse anti-α acetylated tubulin (1:50000, Sigma) and mouse anti-GAPDH (1:8000, Proteintech). After primary antibody incubation, the membranes were treated with HRP-conjugated secondary antibody (Sigma) and developed using an enhanced chemiluminescence kit (Millipore).

### Primary neuronal culture and plasmid transfection

Primary neuronal culture was conducted as described previously^66^. Briefly, round coverslips (VWR) were placed in 24-well plates and coated with poly-L-lysine (Sigma). The cortices of E15.5 wildtype mouse embryos were dissected, trypsinized, and dissociated mechanically into single cell suspension. Cells were seeded at a density of 3 × 10^5^/ml and cultured in Neurobasal medium (Invitrogen) supplemented with 2% B27 (Invitrogen) plus 2 mM glutamine (Sigma) at 37 °C with 5% CO_2_. Twelve hours after plating, tissue debris was removed and the medium was renewed. Neurons at day 7 of culture were transfected with control shRNA or Ulk4 shRNAs combined with GFP (0.5 μg for each) using magnetic beads as per the manufacturer’s instruction (Neuromag, OZ Biosciences, UK). Neurons were fixed at 48 hours after transfection and studied using immunocytochemistry and microscopical examination. Transfection efficiency was determined with the percentage of GFP-positive cells/field and compared between the control and shRNA268 groups by ImageJ. For western blotting, neurons were plated in 6-well plates coated with poly-L-lysine and subject to transfection with either control shRNA or Ulk4 shRNA268 at day 7 of culture. Proteins were extracted after 2 days and subject to western blotting as described above.

### Immunohistochemistry and *in situ* hybridization

Coronal brain sections were prepared from E16.5 mouse brains (20 μm thickness) and P7 brains (40 μm thickness) within a cryostat (Leica 1850). These sections were then processed for immunohistochemistry as described previously^66^. The following primary antibodies were used: rabbit anti-GFP (1:800; Invitrogen), mouse anti-GFP (1:800, Invitrogen), rat anti-BrdU (1:500, Abcam), mouse anti-BrdU (1:200, BD), rabbit anti-Tbr1 (1:500, Abcam), mouse anti-Satb2 (1:500, Abcam), rat anti-Ctip2 (1:500, Abcam) and rabbit anti-cleaved Caspase-3 (1:1000; Cell Signalling). Species-specific Alexa Fluor 488 (1:500, Molecular Probe) or Alexa Fluor 594 (1:1000, Molecular Probe) conjugated secondary antibodies were used to detect primary antibodies. Heat mediated antigen retrieval (citrate buffer, pH = 6.0) was used to reveal BrdU incorporation.

For *in situ* hybridization, mouse brains (E12.5, E15.5, E17.5, P7 and 4 month) were fixed and sectioned at a thickness of 20 μm (for embryos) and 40 μm (for postnatal brains). Sections were mounted on poly-L-lysine coated slides and dried for 2 hours at 55 °C. Riboprobe complementary to 719 bp ULK4 cDNA was transcribed and subject to DIG RNA-labelling (SP6/T7; Roche Diagnostics Ltd. UK). The hybridization was performed as described previously^66^ and the signals were visualized using 5-Bromo-4-chloro-3-indolyl-phosphate (Boehringer Mannheim), and 4-nitroblue tetrazolium salt (BioRad) as substrates for alkaline phosphatase. The sequences of the primers are: Forward, 5′-AACAATCTGGTTGCCTACA-3′; Reverse, 3′-CACTTGAGACTGGTGAGAA-5′.

### Image acquisition and data analysis

All the immunostaining and *in situ* hybridization images were viewed with either a Nikon Eclipse E400 or Zeiss AX10 microscope and sequentially captured by Volocity (5.3.1) or Axiovision (4.8.1). ImageJ was used to define the threshold and to measure the density of fluorescent signals (40X magnification). To reconstruct the dendritic architecture of GFP-positive cells, confocal images were acquired on a Zeiss LSM510 laser scanning confocal microscope using established protocols^67^. Briefly, images were taken with 40x or 63x oil objectives as z series, and around 30–40 images (for 40x) and 40–50 images (for 63x) were taken at 0.6 μm intervals (scan averaged, x4; 512 × 512 pixel resolution) for image reconstruction. The acquisition parameters were kept constant for all scans in the same experiment. Two-dimensional projection reconstructions of z series of images were achieved by using Zeiss Zen software, and morphometric analysis and quantification were carried out using NIH ImageJ programme. *Sholl* analysis for dendritic complexity was carried out by analysing the number of intersections that crossed a series of concentric circles at 10 μm intervals from the cell soma using ImageJ equipped with the *Sholl* Analysis Plugin.

### Statistics

A univariate linear model and post-hoc analysis were run to analyse BrdU incorporation. To compare neurite arborisation pattern and to measure the fluorescent intensity, repeated measurement analysis with post-hoc Tukey test were used to compare between whole groups, followed by multivariate analysis to compare the data of separate distant points among different groups. Data were presented as mean ± S.E.M. *P* < 0.05 was considered statistically significant.

### Study approval

All the animal care and experimental protocols were reviewed and approved by the ethical committees of the Universities of Aberdeen (GM-04-003), Scotland and Tongji (Tongji-med-2009-02), Shanghai. All the experiments were performed in accordance with the relevant guidelines for the care and use of laboratory animals set by both Universities.

## Additional Information

**How to cite this article**: Lang, B. *et al*. Control of cortex development by ULK4, a rare risk gene for mental disorders including schizophrenia. *Sci. Rep.* **6**, 31126; doi: 10.1038/srep31126 (2016).

## Supplementary Material

Supplementary Information
