# Supplementary material for: Control of cortex development by ULK4, a rare risk gene for mental disorders including schizophrenia
Source: Sci Rep. 2016 Sep 27;6:31126. doi: 10.1038/srep31126 (PMC5037360; doi:10.1038/srep31126)
Supplement: Supplementary Information [file srep31126-s1.pdf]

Control of cortex development by ULK4, a rare risk gene for mental disorders including schizophrenia

Bing Lang, Lei Zhang, Guanyu Jiang, Ling Hu, Wei Lan, Lei Zhao, Irene Hunter, Michal Pruski, Ning-Ning Song, Ying Huang, Ling Zhang, David St Clair, Colin D. McCaig, Yu-Qiang Ding

Supplementary Figures and Legends

Supplementary Fig. 1. Hybridization with Ulk4 sense probe produces no specific signals in mouse brain sections.

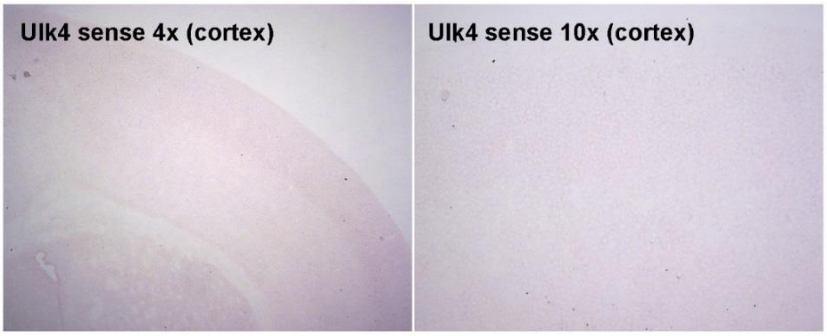

Supplementary Fig. 2. HEK293 cells were lysed and protein was extracted after transfection with panels of shRNAs as described in the section of Method. Western blotting using anti-c-Myc antibody also demonstrates similar order of knockdown capacity among the three Ulk4 shRNAs (268>269>270). N=3, * $p < 0.05$, ** $p < 0.01$.

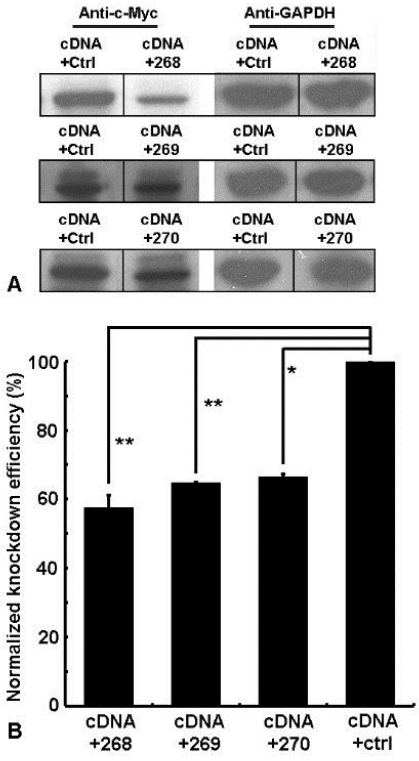

Supplementary Fig.3. The expression of Ulk4 Immunostaining is demonstrated in E16.5 brain sections after *in utero* electroporation. In the control group (A,D,G), most of GFP-postive cells (contained control shRNA) express Ulk4 strongly (in red). However, these cells only display very weak Ulk4 in shRNA268 (B,E,H) or shRNA269 groups (C,F,I). Representive cells are indicated with white arrowheads. Bar=20µm.

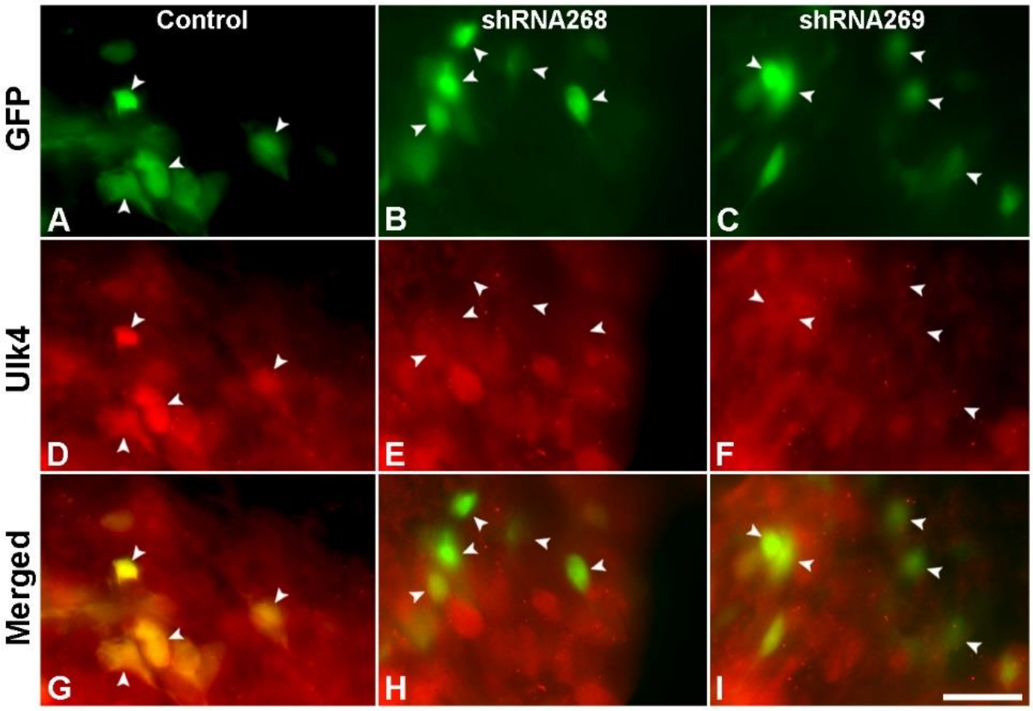

Supplementary Fig.4. Representative images show the BrdU/GFP double-labeled cells (white arrows) at E15.5 of control (A), shRNA268 (B) and shRNA269 groups (C). Bars= 50µm.

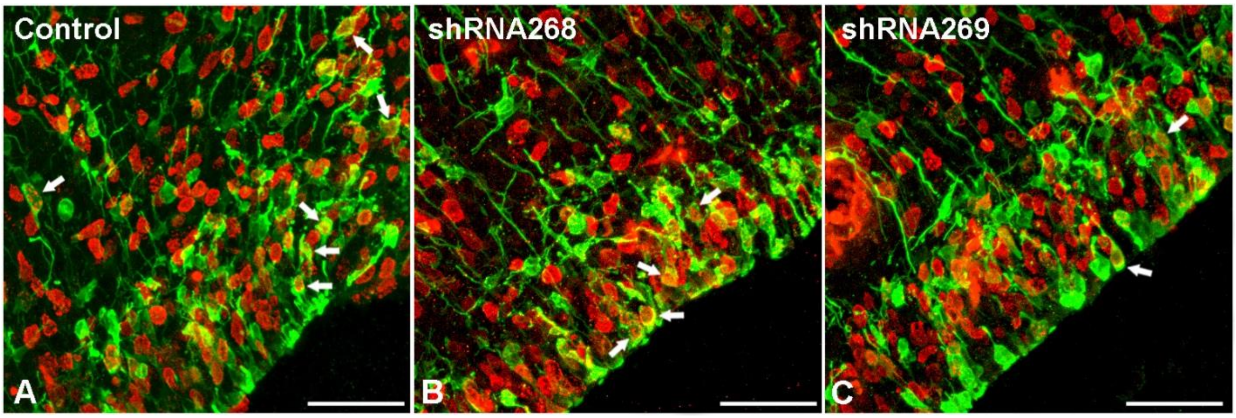

Supplementary Fig. 5. Immunodouble-labeling shows that neurons integrated with shRNA268 (GFP-positive) in the cortical superficial layers express Satb2 (in red, A,D,G,J), but not Ctip2 (in red, B,E,H,K) or Tbr1 (in red, C,F,I,L). Representative double-labelled cells are indicated with white arrows. Single-labelled GFP or Ctip2 cells are indicated with white arrowheads or narrow arrows respectively. Bars=100 µm in A-C, 20µm in D-L.

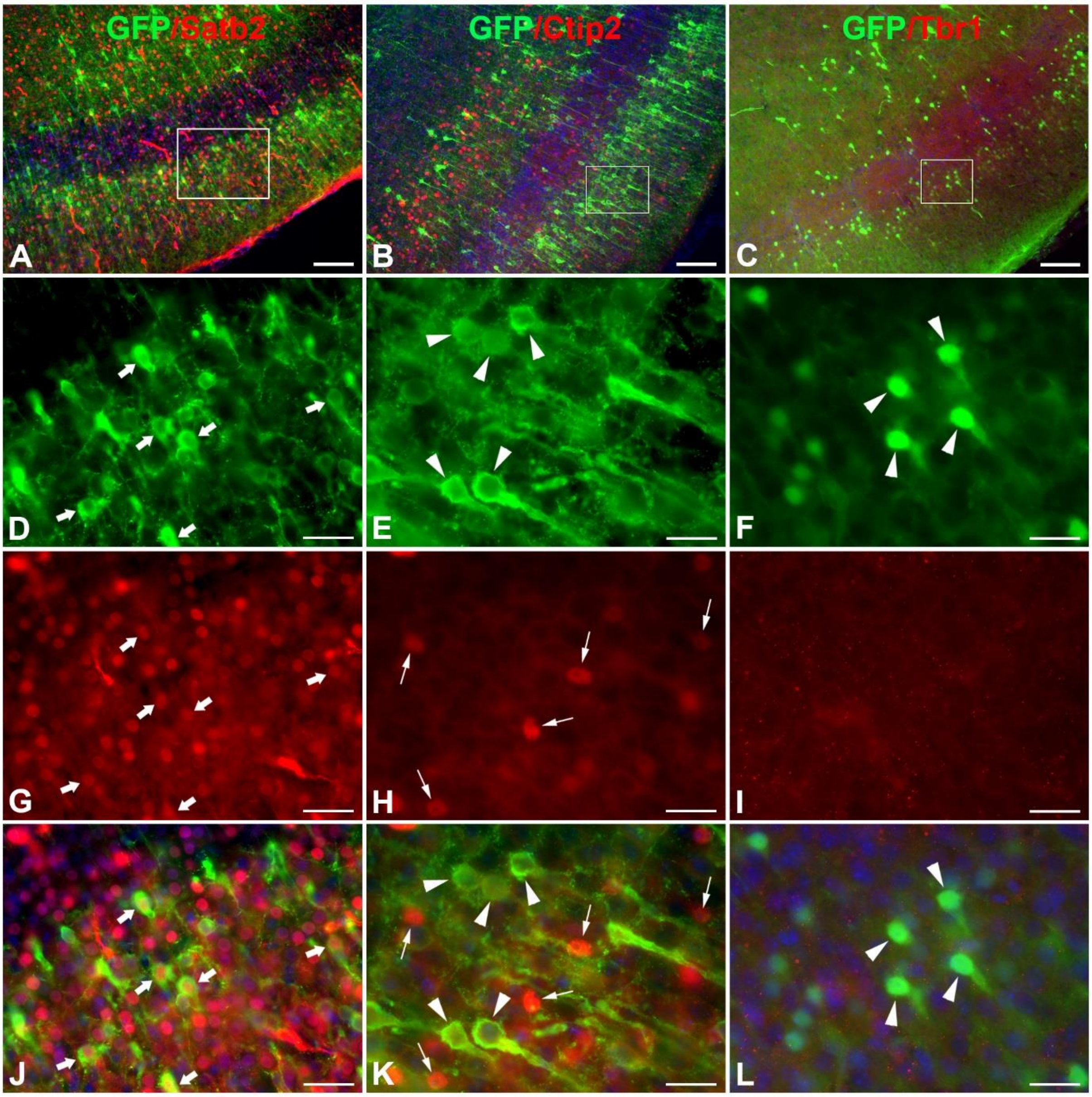

Supplementary Fig. 6. Imaging analysis reveals that silencing of Ulk4 in both cultured neurons (A) and cortical sublayers (B) leads to reduced fluorencent intensity of acetylated α tubulin. * *p*<0.05; ** *p*<0.01.

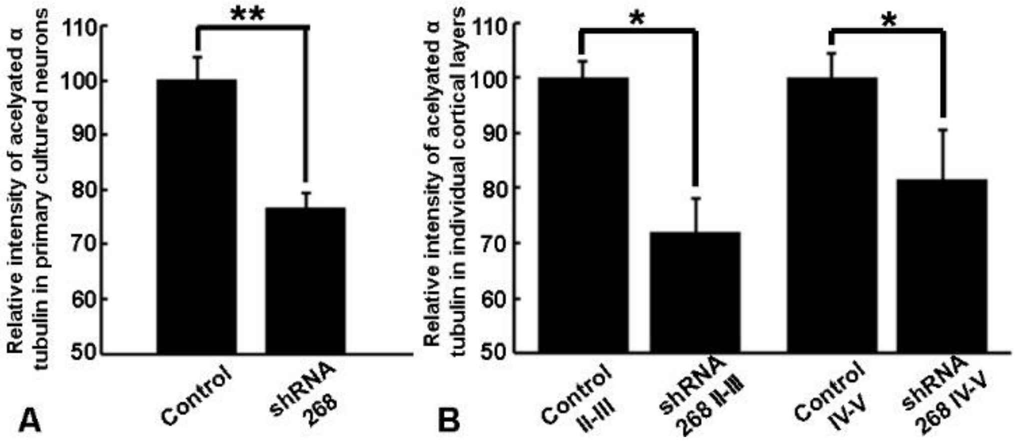

Supplementary Fig. 7. E15.5 embryos are electroporated with shRNA269 and their brains are collected at P7 for immunostaining with anti-acetylated α tubulin. Note that neurons with Ulk4 knockdown (GFP-positive) also express decreased α acetylated tubulin. Bars=25μm.

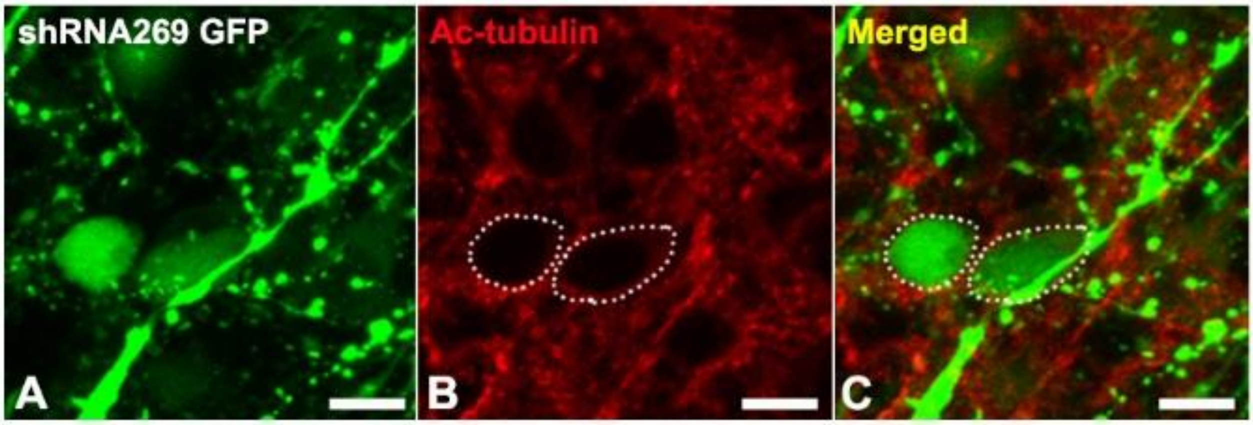

The full-length blots of shRNA identification are listed as below

Identification of shRNA268:

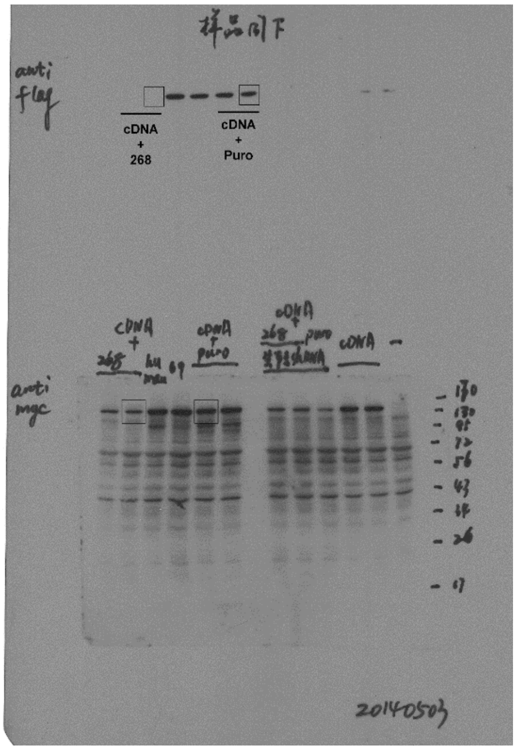

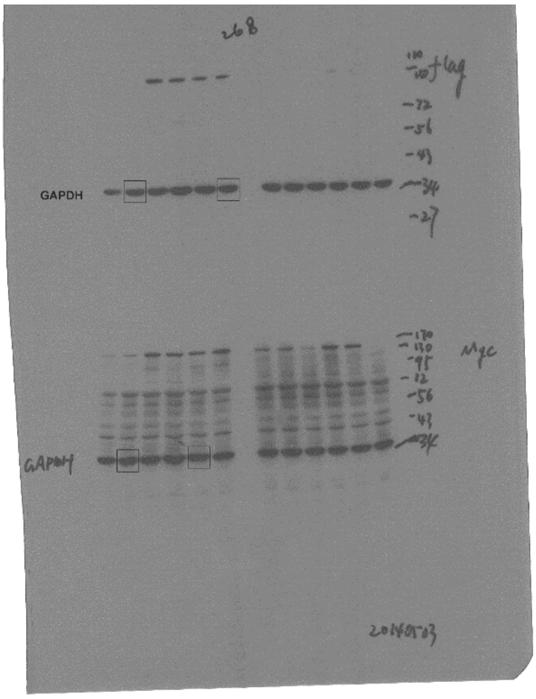

Left:

Representative electrophoresis shows that shRNA268 effectively inhibits Ulk4 expression in HEK293 cells.

Top gel: anti-flag blot. Two boxed bands (left, shRNA268; right, control) are cropped and presented in Fig. 2.

Bottom gel: anti-c-Myc blot. Two boxed bands (left, shRNA268; right control) are cropped and presented in Fig. S2.

Right:

Anti-GAPDH immunoblotting reveals similar protein loading.

Top gel: Two boxed bands, which correspond to the two boxed bands in anti-flag blot (top gel in left), are cropped and presented in Fig. 2.

Bottom gel: Two boxed bands, which correspond to the two boxed bands in anti-c-Myc blot (bottom gel in left), are cropped and presented in Fig. S2.

Identification of shRNA269 and shRNA270:

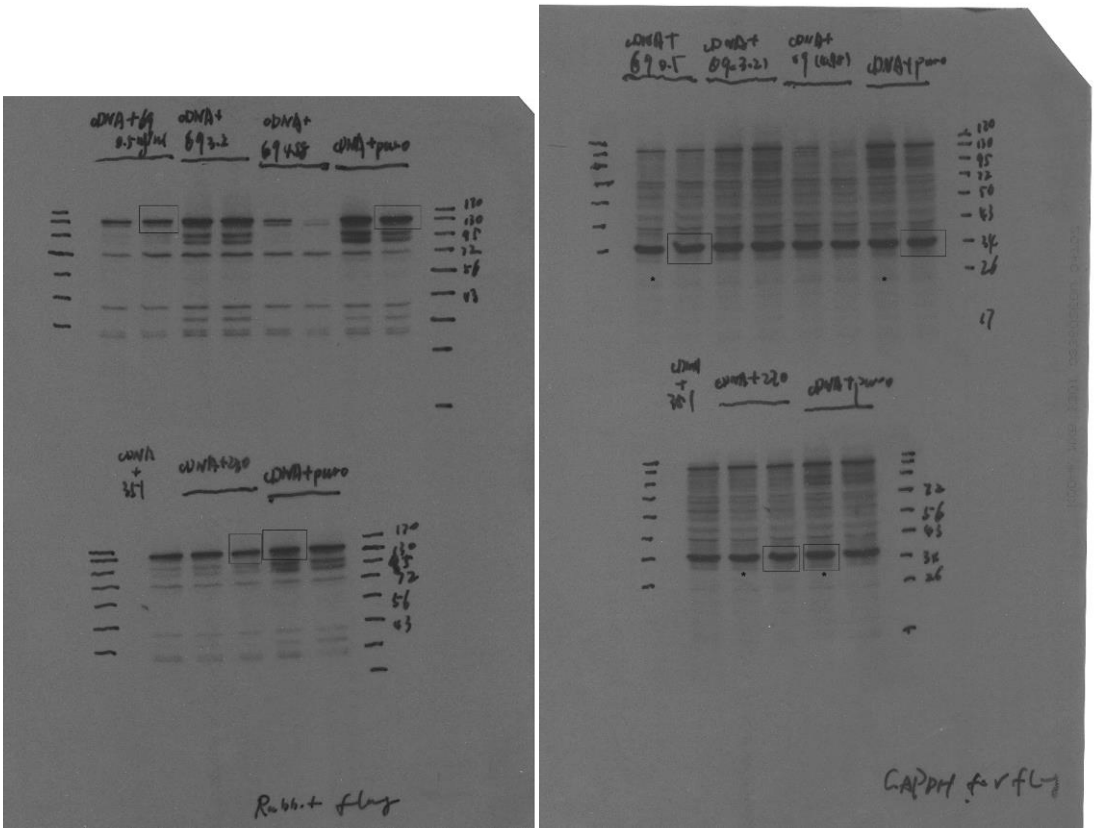

Left:

The representative blotting of anti-flag reveals different knockdown effects of shRNA269 (top gel) and shRNA270 (bottom gel).

Top gel: Two boxed bands (left, shRNA269; right, control) are cropped and presented in Fig. 2.

Bottom gel: Two boxed bands (left, shRNA270; right, control) are cropped and presented in Fig. 2.

Right:

Anti-GAPDH immunoblotting reveals similar protein loading.

Top gel: Two boxed bands, which correspond to the two boxed bands in anti-flag blot (shRNA269; top gel), are cropped and presented in Fig. 2.

Bottom gel: Two boxed bands, which correspond to the two boxed bands in anti-flag blot (shRNA270; bottom gel), are cropped and presented in Fig. 2.

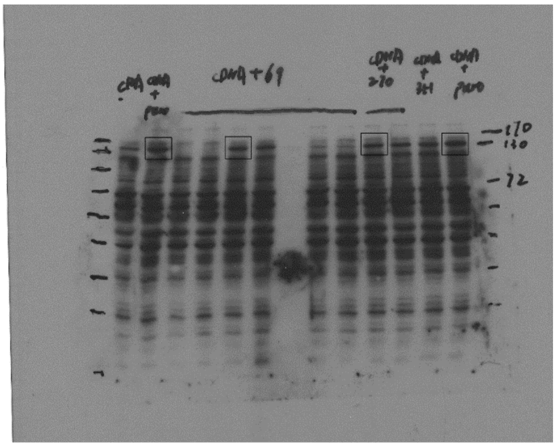

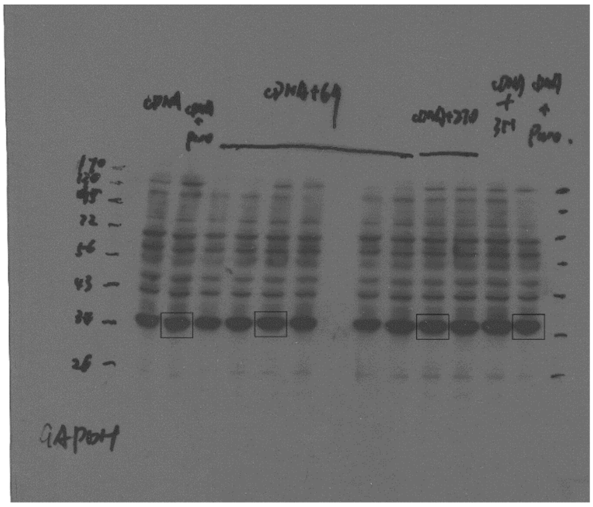

Left:

The representative blotting of anti-c-Myc reveals different knockdown effects of shRNA269 and shRNA270.

Lanes 1-8: Two boxed bands (left, control; right, shRNA269) are cropped and presented in Fig. S2.

Lanes 9-12: Two boxed bands (left, shRNA270; right, control) are cropped and presented in Fig. S2.

Right:

Anti-GAPDH immunoblotting reveals similar protein loading.

Lanes 1-8: Two boxed bands, which correspond to the two boxed bands in lanes 1-8 (anti-c-Myc, shRNA268, left), are cropped and presented in Fig. S2.

Lanes 9-12: Two boxed bands, which correspond to the two boxed bands in lanes 9-12 (anti-c-Myc, shRNA360, left), are cropped and presented in Fig. S2.
